# Macronutrient Intake and Carbohydrate Quality in Indian Adults With and Without Type 2 Diabetes: The Multicentre I-STARCH-1 Cross-Sectional Study

**DOI:** 10.3390/nu18182993

**Published:** 2026-09-13

**Authors:** Nitin Kapoor, Sanjay Kalra, Neeta Deshpande, Sheryl S. Salis, Smriti Gadia, Thamburaj Anthuvan

**Affiliations:** 1 Department of Endocrinology, Diabetes and Metabolism, Christian Medical College, IDA Scudder Road, Vellore 632004, Tamil Nadu, India; nitin.endocrine@gmail.com; 2 Department of Endocrinology, Bharti Hospital, Karnal 132001, Haryana, India; brideknl@gmail.com; 3 Belgaum Diabetes Centre and CentraCare Super Speciality Hospital and Medical Research Centre, 241 Devaki, Ranade Road, Tilakwadi, Belagavi 590006, Karnataka, India; neetarohit@gmail.com; 4 Nurture Health Solutions, Mumbai 400057, Maharashtra, India; sheryl@nurturehealthsolutions.com; 5 Scientific Services, USV Private Limited, Arvind Vithal Gandhi Chowk, BSD Marg, Station Road, Govandi East, Mumbai 400088, Maharashtra, India; smriti.gadia@usv.in; 6 MAHE School of Business, Manipal Academy of Higher Education, Dubai International Academic City, Dubai P.O. Box 345050, United Arab Emirates; 7 Corporate Strategy, USV Private Limited, Arvind Vithal Gandhi Chowk, BSD Marg, Station Road, Govandi East, Mumbai 400088, Maharashtra, India

**Keywords:** macronutrient intake, carbohydrate composition, dietary fibre, mono- and disaccharides, type 2 diabetes mellitus, dietary patterns, India, cross-sectional study

## Abstract

**Background**: Contemporary data on macronutrient intake and carbohydrate composition in Indian adults are limited. The Indian Study to Assess Real-World Carbohydrate Consumption (I-STARCH-1) characterised these patterns among adults with and without type 2 diabetes mellitus (T2DM). **Methods**: This multicentre cross-sectional study included 1104 adults (690 with T2DM and 414 without) recruited by convenience sampling from 29 diabetes-focused centres across 14 Indian states. Dietary intake was assessed using a structured three-day dietary recall. Between-group differences were estimated using linear mixed-effects models adjusted for age and sex, with the centre as a random intercept. **Results**: Carbohydrates contributed 62.1% of the energy, fat 25.1%, and protein 12.8%. Mono- and disaccharides accounted for 20.1% of the total carbohydrate intake, and fibre intake averaged 15.4 g/day. Participants with T2DM had lower carbohydrate %E and a lower simple-carbohydrate proportion of total carbohydrate, but higher protein and fat %E; these differences persisted after adjustment. Absolute fibre intake did not differ between the groups. The composition varied modestly across the four geographic zones. Descriptively, the carbohydrate contribution was lower and the fat contribution was higher than that in the Study To Assess the dietaRy CarboHydrate content of the Indian type 2 diabetes population (STARCH) 2014 cohort, but the cohorts were non-harmonised. **Conclusions**: Carbohydrates remained the predominant energy source, with low fibre intake. The overall macronutrient profile closely resembled the contemporary national population estimates. The findings support attention to carbohydrate composition and fibre adequacy alongside quantity but describe healthcare-seeking adults rather than the general Indian population.

## 1. Introduction

India’s nutrition transition is occurring alongside a substantial burden of type 2 diabetes mellitus (T2DM) and continuing changes in dietary patterns and food environments [1]. Dietary carbohydrates remain a major source of energy in Indian diets, but their metabolic implications depend not only on quantity but also on food source, fibre content, degree of processing, and glycaemic characteristics [2,3,4]. Contemporary national evidence indicates that carbohydrates continue to contribute a large proportion of the total energy intake in Indian adults, with refined cereals accounting for a substantial share [5]. T2DM represents a major public health challenge in India, with an estimated 101 million adults living with diabetes and 136 million with prediabetes [6].

Dietary patterns across India are heterogeneous, reflecting differences in staple foods, culinary practices, socioeconomic circumstances, and food access [7,8]. Nevertheless, cereal-based carbohydrates remain prominent in many Indian diets [9], while dietary guidance increasingly emphasises balanced macronutrient intake, fibre-rich foods, and consideration of carbohydrate source and composition [8,10,11]. Concurrently, urbanisation, rising incomes, and expanding access to packaged foods, ready-to-eat products, and sugar-sweetened beverages have altered the food environment in which these diets are consumed [12,13]. These features underscore the need for contemporary multicentre data characterising macronutrient intake and carbohydrate composition among Indian adults, including comparisons between those with and without T2DM.

The STARCH (Study To Assess the DietaRy CarboHydrate content of the Indian type 2 diabetes population), published in 2014, was an important multicentre survey describing macronutrient intake among Indian adults with and without T2DM [14]. Carbohydrates were the predominant source of dietary energy in that cohort, providing an important historical benchmark for subsequent Indian dietary research. However, STARCH 2014 and the Indian Study to Assess Real-World Carbohydrate Consumption (I-STARCH-1) are independent cross-sectional studies with differences in sampling, eligibility criteria, dietary assessment, and geographic framework [15]. Therefore, the earlier study was used here as a descriptive historical reference rather than a longitudinal comparator. More broadly, changes in food availability, urbanisation and dietary practices reinforce the need for contemporary data without presupposing the direction or magnitude of any difference from earlier cohorts [1,16].

Recent Indian evidence has further refined our understanding of carbohydrate intake by demonstrating the importance of food sources and overall macronutrient balance. National analyses have shown substantial contributions from refined cereals and suggested that metabolic associations depend on both the amount and source of carbohydrate consumed [5]. Glycaemic index profiling of traditional Indian foods likewise demonstrates considerable heterogeneity among carbohydrate-containing foods that cannot be captured by carbohydrate quantity or polymer length alone [3]. Data-driven modelling of Indian dietary data has also been used to derive population-specific macronutrient targets for the prevention and remission of diabetes [17]. Therefore, contemporary assessment requires consideration of macronutrient distribution and dietary fibre and carbohydrate composition while recognising the limitations of simple-versus-complex carbohydrate classification.

Accordingly, the primary objective of I-STARCH-1 was to characterise macronutrient composition among Indian adults with and without T2DM, including the contributions of carbohydrate, protein and fat to total energy intake and dietary fibre intake. Secondary objectives were to evaluate the relative proportions of simple and complex carbohydrates and to describe meal frequency, water intake and biochemical markers. Comparisons by T2DM status accounted for clustering within participating centres. Comparisons with published summary statistics from the independent STARCH 2014 cohort were undertaken solely for historical context. Geographic-zone analyses and the investigator-derived Diet-Quality Index (DQI) and Protein–Fat Quality Index (PFQI) were exploratory. We hypothesised that the primary dietary measures would differ by T2DM status. 

## 2. Methodology

### 2.1. Study Design and Setting

I-STARCH-1 was a multicentre, cross-sectional observational study conducted in 2025 across 29 diabetes-focused healthcare centres in 14 Indian states, spanning the north, south, east, and west geographic zones. The centres were led by practising diabetologists or physicians (62.1%), endocrinologists (34.5%), and dietitians (3.4%). Participants were recruited by convenience sampling from these centres; no community or other non-clinical settings contributed participants to the final analysed cohort. Therefore, the study sample should be interpreted as a healthcare-seeking, clinic-based cohort and not as representative of the general Indian population. All centres followed standardised procedures for recruitment, dietary assessment, investigator training, and data quality assurance under central coordination. The final analytic sample comprised 1104 adults, including 690 with T2DM and 414 without T2DM. The study protocol was published previously [15]. Reporting followed the Strengthening the Reporting of Observational Studies in Epidemiology (STROBE) statement and its nutritional epidemiology extension (STROBE-nut) [18,19]; a completed checklist is provided in Supplementary Table S1.

### 2.2. Ethical Approval

The study was approved by the Institutional Ethics Committee of Shah Lifeline Hospital & Heart Institute (approval no. SLH-IEC/2025/006; approved on 17 January 2025). It was conducted in accordance with the Declaration of Helsinki, the Indian Council of Medical Research National Ethical Guidelines for Biomedical and Health Research Involving Human Participants, and International Council for Harmonisation Good Clinical Practice guidelines. Written informed consent was obtained from all participants before enrolment. As I-STARCH-1 was a non-interventional, cross-sectional observational study involving no investigational drug or intervention, clinical-trial registration and regulatory approval under the New Drugs and Clinical Trials Rules, 2019, were not applicable. 

### 2.3. Participants and Eligibility

Stratification by T2DM status was central to the study’s primary objective of characterising macronutrient composition and dietary fibre intake in adults with and without diabetes. Adults aged ≥ 18 years, with or without T2DM, were recruited from participating healthcare centres across 14 states. No upper age limit was specified; the observed range was 19–88 years (mean age, 49.8 ± 11.9 years). All the participants provided written informed consent. Eligibility required that relevant clinical documentation from the preceding three months was available for assessment; this did not require recent fasting plasma glucose, post-prandial glucose or HbA1c values, since in these real-world settings documentation commonly comprised previous prescriptions and paper-based consultation records rather than uniformly available electronic records or laboratory reports. Participants with type 1 or secondary diabetes and those meeting other protocol-defined exclusion criteria were not enrolled. The detailed inclusion and exclusion criteria are listed in Table 1. The final analytic sample comprised 1104 adults (690 with T2DM and 414 without T2DM).

### 2.4. Definition of T2DM

T2DM status was recorded by the site investigator using a case report form. A pre-existing physician diagnosis documented in the available clinical records, including previous prescriptions, was the principal basis for classification as T2DM. Where formal diagnostic documentation was unavailable, a participant-reported physician diagnosis was considered, together with current glucose-lowering medication use. Classification as non-T2DM required the absence of any known or documented history of diabetes and no current use of glucose-lowering medications. Fasting plasma glucose < 126 mg/dL or HbA1c < 6.5% supported non-T2DM classification where such values were available; however, recent biochemical values were not available for participants in the non-T2DM group. Therefore, biochemical confirmation was not mandatory, and no study-specific diagnostic testing was performed. The possibility of undiagnosed diabetes among participants classified as non-T2DM cannot be excluded and is acknowledged as a limitation of this study. Participants with type 1 and secondary diabetes were excluded on clinical grounds.

### 2.5. Recruitment and Participant Flow

**Figure 1:**
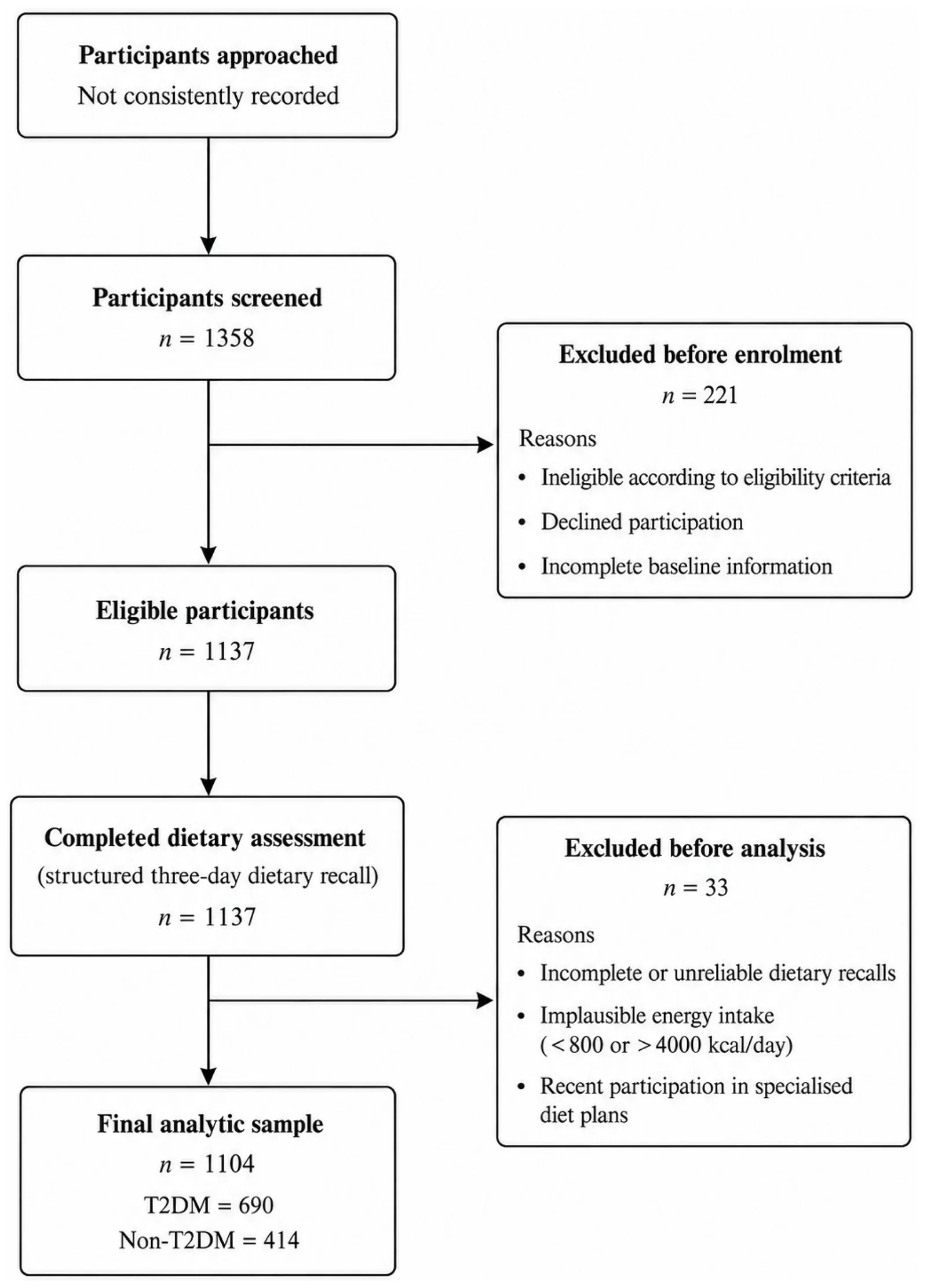
Participant recruitment and selection flow diagram for the I-STARCH-1 study.

Participants were recruited from 29 healthcare centres in 2025 using convenience sampling. The published protocol specified a pragmatic target of approximately 30 participants with T2DM and 10 without T2DM per centre. These targets were not achieved uniformly; the final analytic cohort comprised 690 participants with T2DM (62.5%) and 414 without T2DM (37.5%). The T2DM proportion therefore reflects intentional recruitment enrichment together with site-level recruitment patterns and should not be interpreted as population prevalence. The number of individuals approached before screening was not consistently recorded; therefore, a conventional response rate could not be calculated. Of the 1358 individuals screened, 1137 were enrolled, and 1104 remained in the final analytic sample after data quality exclusions. The analytic sample was distributed across centres, with a median of 39 participants (range, 17–50). Recruitment and selection are summarised in Figure 1; disaggregated counts by exclusion reason were not recorded.

### 2.6. Sample Size Determination

A formal a priori sample size or power calculation was not performed. Instead, the study adopted a pragmatic recruitment strategy based on the protocol target of approximately 40 participants per centre, comprising approximately 30 with T2DM and 10 without T2DM, to achieve broad geographic coverage and deliberate enrichment of participants with T2DM. Of the 1137 enrolled participants, 1104 were retained in the final analytic dataset after data-quality exclusions. For carbohydrate contribution, one of the primary dietary outcomes, the observed mean was 62.1% of total energy (SD 5.4), corresponding to a 95% confidence interval of approximately ±0.3 percentage points around the cohort mean. This interval describes the observed precision and was not used as an a priori sample-size justification. 

### 2.7. Dietary Assessment

Dietary intake was assessed using a structured three-day dietary recall administered face-to-face by trained investigators using a standardised multi-pass technique and household measures. Before study implementation, investigators and study personnel across participating centres received standardised training in dietary-recall collection and portion recording, using a measuring set comprising 1, ½, ⅓ and ¼ cup measures and 15, 7.5, 5 and 2.5 mL measuring spoons, with cooked portion quantities recorded throughout. The recalls were recorded using a structured dietary survey questionnaire developed for the study (Supplementary File S1), with intake documented separately for Days 1, 2 and 3. Calendar dates and days of the week were not captured, and no fixed schedule of consecutive or non-consecutive days, or weekdays and weekend days, was prescribed by the study protocol. Field supervisors reviewed the recalls for completeness and conducted random verification calls to resolve discrepancies. Dietary variables represented the average intake across the three recorded recall days.

### 2.8. Food Composition and Nutrient Coding

Reported foods and beverages were processed using a custom computational food-matching tool developed for the study. The tool applied fuzzy-string matching to compare standardised food descriptions from participant recalls with entries in the Indian Nutrient Databank (INDB) [20], whose food-composition data are primarily derived from the Indian Council of Medical Research–National Institute of Nutrition (ICMR–NIN) Indian Food Composition Tables 2017 [21], with gaps supplemented from the 2004 Indian tables [22] and United Kingdom and United States nutrient databases. The tool was not a generative or deep-learning model. Candidate food matches were ranked by text similarity; the similarity score was used only for ranking and did not determine the final assignment. No food code was accepted on the basis of the similarity score alone, and all candidate assignments required confirmation by an investigator or qualified dietitian.

The workflow comprised four steps. First, reported food descriptions were standardised. Second, household portion measures recorded in the case report form, including a standard cup volume of 200 mL, bowls, item counts and fractional portions, were converted algorithmically to grams or millilitres using food-specific unit or volume equivalents held within the coding workflow; fractional portions were converted proportionally, and no published portion-size atlas was used. Third, standardised descriptions were matched to candidate INDB food codes and reviewed before final coding. Mixed dishes were decomposed to ingredient level using reported recipe and preparation information, with ingredient assignments reviewed where required. For regional, ambiguous and branded items without an exact database match, candidate matches were adjudicated using participant-reported ingredients and preparation methods, ingredient-level reconstruction or, where necessary, the nearest nutritionally comparable database entry. Fourth, nutrient values were calculated from the confirmed food mappings. String matching, portion conversion and ingredient-level decomposition were automated, whereas final food-code confirmation and adjudication of ambiguous matches were performed manually. A random subset of algorithmically converted portions was independently cross-checked by qualified dietitians, and discrepancies were resolved before nutrient computation. Neither the implementation-level similarity function nor the original provenance of the food-specific gram-equivalent values was retained in the final study documentation, and no numerical acceptance threshold governed final coding.

### 2.9. Macronutrient Computation

Daily energy and nutrient intakes were calculated from confirmed food mappings using the INDB [20]. Carbohydrate, protein and fat intakes were expressed in g/day, and their energy contributions were calculated using factors of 4, 4 and 9 kcal/g, respectively [23], and expressed as %E. Simple carbohydrate comprised mono- and disaccharides identified at the food-code level, including intrinsic and added sugars where represented in the database. Intrinsic, added, and free sugars could not be distinguished separately. Complex carbohydrates were calculated as total carbohydrates minus simple carbohydrates. Both were derived from continuous participant-level nutrient values and expressed as percentages of total carbohydrate intake, summing up to 100%. Dietary fibre was obtained directly from the food composition database and reported as g/day and g/1000 kcal. Reported carbohydrate, protein and fat %E used a fibre-exclusive energy denominator. For the exploratory DQI, protein, fibre and simple carbohydrate were each expressed as percentages of the same fibre-inclusive energy denominator, assigning 2 kcal/g to fibre; these DQI-specific percentages were separate from the reported macronutrient %E values.

### 2.10. Anthropometric Measures

Body mass index (BMI) was calculated as weight in kilograms divided by height in metres squared. Waist circumference was recorded as an estimated measure rather than a centrally verified direct tape measurement and was therefore reported descriptively as the estimated waist circumference. It was not included as a covariate in the adjusted analyses, and its measurement limitations are acknowledged in the limitations section.

### 2.11. Study Outcomes

The primary dietary outcomes were carbohydrate, protein and fat contributions to total energy intake (%E) and dietary fibre intake (g/day; additionally reported as g/1000 kcal). Secondary outcomes were simple and complex carbohydrate proportions (% of total carbohydrate), meal frequency, water intake and biochemical markers. Dietary outcomes were compared between participants with and without T2DM. Geographic-zone comparisons and analyses of the investigator-derived DQI and Protein–Fat Quality Index (PFQI) were exploratory; both indices are unvalidated. Comparisons with published STARCH 2014 summary statistics [14] were descriptive and undertaken solely for historical context. 

### 2.12. Diet-Quality Index (DQI) and Protein–Fat Quality Index (PFQI)

The DQI and PFQI are investigator-derived exploratory composite measures informed by established diet-quality frameworks [24,25]; neither has been externally validated. The DQI was calculated at the participant level as protein (%E) + fibre (%E) − simple carbohydrate (%E). All three DQI components used the same fibre-inclusive energy denominator, with fibre assigned 2 kcal/g, as described in Section 2.9. PFQI was calculated at the participant level as protein (%E) divided by fat (%E). Higher DQI values indicate a more favourable balance of the component nutrients, and higher PFQI values indicate a greater relative contribution of protein to fat. No established clinical thresholds exist, and both indices were interpreted descriptively. Because PFQI was computed as the mean of participant-level ratios, it is not mathematically equivalent to the ratio of cohort mean protein to cohort mean fat, and the two quantities are not interchangeable when comparing cohorts.

### 2.13. Data Quality Assurance and Missing Data

Data were reviewed centrally for completeness, internal consistency, and implausible values before analysis, with energy intake checked against a plausibility range of 800–4000 kcal/day. Source verification identified and corrected three transcription errors in total energy; the final analytic range was 1000–2919 kcal/day, with no further exclusions. Twenty-seven HbA1c values recorded as proportions were rescaled to percentage units, laboratory fields were standardised for formatting and units, and simple and complex carbohydrate percentages were recomputed from continuous participant-level values (Supplementary File S2). Primary dietary outcomes were complete for all 1104 participants. Missingness was substantial for fasting plasma glucose (43.5%), post-prandial glucose (47.1%), and HbA1c (43.9%). At least one biochemical value was available for 643 of 690 participants with T2DM (93.2%), whereas none was available for the 414 participants without T2DM; availability also varied by centre and geographic zone (Supplementary Table S3). Biochemical data availability was strongly related to T2DM status, and no biochemical values were available in the non-T2DM group; therefore, these data were not imputed and were reported descriptively only.

### 2.14. Statistical Analysis

Continuous variables were summarised as mean ± standard deviation or median (interquartile range), as appropriate, and categorical variables as frequencies and percentages. Unadjusted comparisons between participants with and without T2DM and across geographic zones used independent-samples *t*-tests or one-way analysis of variance (ANOVA), Mann–Whitney U or Kruskal–Wallis tests for non-normally distributed variables, and chi-square tests for categorical variables. To account for the multicentre design and potential confounding, linear mixed-effects models with participating centre as a random intercept were fitted by restricted maximum likelihood for five dietary outcomes: carbohydrate %E, protein %E, fat %E, simple carbohydrate as a percentage of total carbohydrate, and fibre intake (g/day). The primary model included T2DM status, age, and sex as fixed effects; the sensitivity model additionally included restricted diet status, diet type, and exercise status. The age- and sex-adjusted model was designated as primary because the behavioural covariates may themselves be influenced by diabetes diagnosis and subsequent dietary modification. Adjusted mean differences are reported as T2DM minus non-T2DM with 95% confidence intervals, together with centre-level intraclass correlation coefficients (Supplementary Table S2). The adjusted estimates describe the differences between the sampled groups and do not imply an effect of T2DM on dietary intake. The DQI and PFQI analyses were exploratory, no formal correction for multiple comparisons was applied, and secondary and exploratory findings were interpreted cautiously. Analyses were performed using IBM SPSS Statistics for Windows, Version 29.0 (IBM Corp., Armonk, NY, USA), with mixed-effects models fitted in Python 3.12 using the statsmodels 0.15.0 package. Statistical significance was set at *p* value < 0.05.

### 2.15. Protocol Deviations

Several deviations from the published protocol occurred during implementation. Of the 33 planned centres, 29 recruited participants; four sites did not proceed because of feasibility constraints and competing professional commitments. The final cohort was recruited from diabetes-focused healthcare centres. The planned urban versus semi-urban comparison was replaced by a comparison across four centre-based geographic zones, because centre location did not reliably indicate whether participants lived in urban or semi-urban areas. Planned multivariable and sensitivity analyses were performed using centre-adjusted mixed-effects models. Primary dietary outcomes were complete and required no imputation, whereas biochemical data were not imputed or compared between groups because they were entirely unavailable in the non-T2DM group.

## 3. Results

### 3.1. Baseline Characteristics

The study included 1104 participants, comprising 690 with T2DM (62.5%) and 414 without T2DM (37.5%); this distribution reflects the prespecified recruitment enrichment described in Section 2.5. Participants with T2DM were older, had greater estimated waist circumference, and more frequently reported a restricted diet during the preceding year than those without T2DM (each *p* < 0.001). BMI and the distributions of sex, diet type, exercise participation, and alcohol consumption did not differ between groups; the geographic-zone distribution did not differ significantly between groups (*p* = 0.055). Because the estimated waist circumference was not obtained by direct measurement, the between-group difference in this variable is reported descriptively only. The detailed characteristics are presented in Table 2.

### 3.2. Energy and Macronutrient Intake

**Figure 2:**
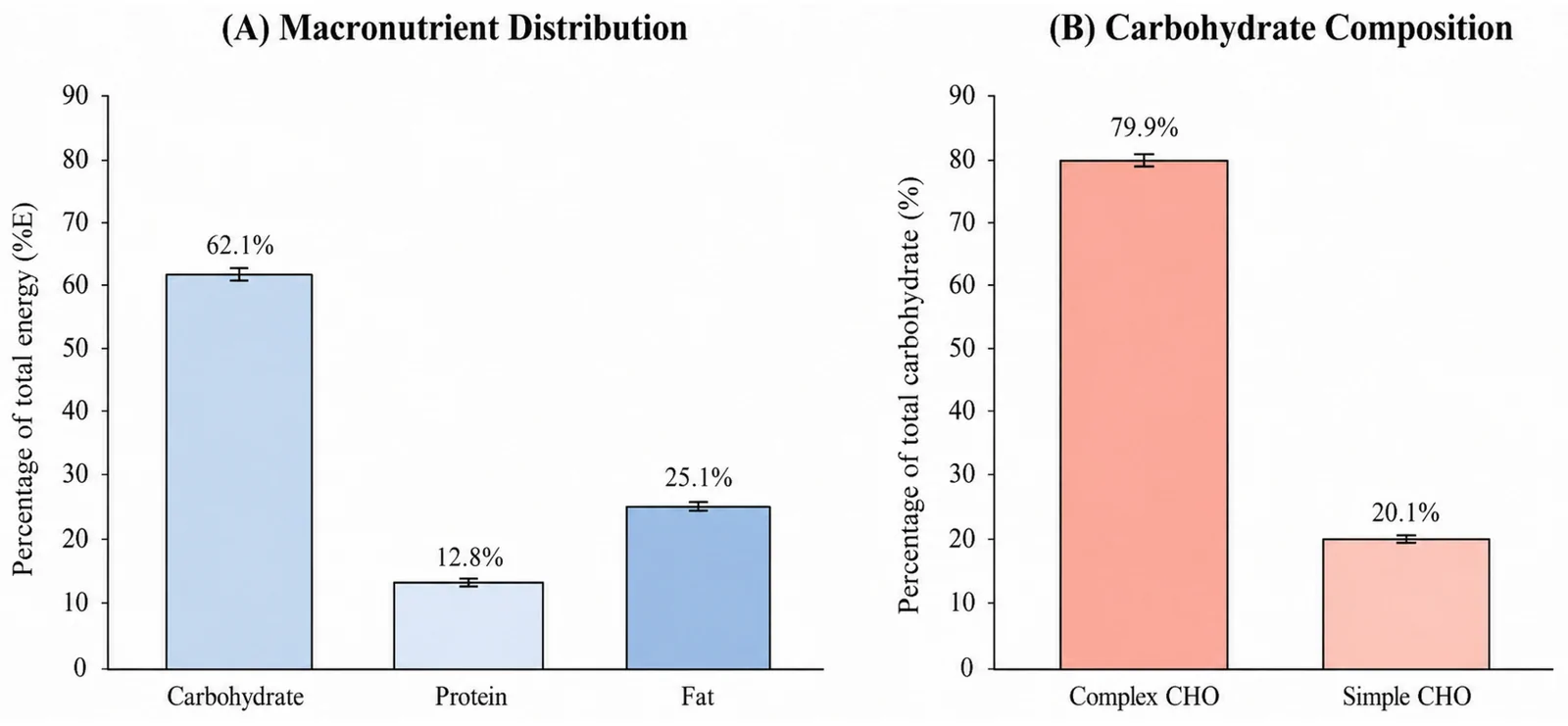
Overall macronutrient distribution and carbohydrate composition in the I-STARCH-1 cohort. (**A**) Mean percentage contributions of carbohydrate, protein and fat to total energy intake among all participants (*N* = 1104). (**B**) Mean proportions of simple and complex carbohydrate expressed as percentages of total carbohydrate intake. Error bars represent 95% confidence intervals. Note: Values represent cohort means derived from a structured three-day dietary recall. Macronutrient contribution is expressed as percentage of total energy intake (%E). Simple carbohydrate comprises mono- and disaccharides identified at the food-code level and does not represent free-sugar intake. Simple and complex carbohydrate sum to 100% of total carbohydrate intake. Abbreviations: CHO, carbohydrate; %E, percentage of total energy intake.

The mean daily energy intake was 1778.6 ± 369.9 kcal/day. Carbohydrate was the predominant energy source, contributing 62.1 ± 5.4%E, followed by fat (25.1 ± 5.5%E) and protein (12.8 ± 3.5%E) (Figure 2A). The mean dietary fibre intake was 15.4 ± 3.2 g/day, equivalent to 9.1 ± 2.8 g/1000 kcal. Reported intakes represent the averages across the three recall days and may be affected by recall-related under-reporting. Simple and complex carbohydrates accounted for 20.1 ± 6.5% and 79.9 ± 6.5% of the total carbohydrate intake, respectively (Figure 2B).

The profiles according to T2DM status are presented in Table 3. Simple carbohydrates represent mono- and disaccharides identified at the food-code level and should not be interpreted as free sugar intake.

### 3.3. Adjusted Differences by T2DM Status

After adjustment for age and sex and accounting for clustering by participating centre, participants with T2DM had a lower carbohydrate contribution to total energy by 3.23 percentage points (95% CI, −3.82 to −2.63) and a lower simple-carbohydrate proportion of total carbohydrate by 4.15 percentage points (95% CI, −4.87 to −3.43) than those without T2DM. Protein and fat contributions were higher by 0.82 (95% CI, 0.41–1.24) and 2.38 (95% CI, 1.76–2.99) percentage points, respectively. Absolute dietary fibre intake did not differ after adjustment (0.28 g/day; 95% CI, −0.07 to 0.64; *p* = 0.118). Centre ICCs ranged from 0.130 to 0.201, indicating modest clustering. Additional adjustment for restricted-diet status, diet type and exercise produced materially similar estimates; the corresponding adjusted differences and 95% confidence intervals are reported in Supplementary Table S2. Unadjusted, primary adjusted and sensitivity-model estimates are presented in Supplementary Table S2. These estimates describe differences between the sampled groups and do not imply an effect of T2DM on dietary intake.

### 3.4. Geographic Distribution of Macronutrient Intake

Geographic-zone comparisons were exploratory analyses across the North (*n* = 220), South (*n* = 328), East (*n* = 343), and West (*n* = 213) zones (Table 4). Carbohydrate remained the predominant energy source in all zones, contributing 61.5 to 63.2%E, followed by fat (22.9 to 26.6%E) and protein (11.9 to 13.9%E). Simple carbohydrates contributed 19.3–21.3% and complex carbohydrates 78.7–80.7% of total carbohydrate intake. Total energy intake did not differ across zones (*p* = 0.777), whereas carbohydrate, protein, and fat contributions, dietary fibre, and carbohydrate composition differed overall (all *p* < 0.001). The absolute differences were generally modest. These analyses were unadjusted exploratory comparisons, with no correction for multiple comparisons or pairwise testing, and should therefore be interpreted descriptively rather than as population-level geographic differences.

### 3.5. Biochemical Data Availability and Descriptive Findings

Biochemical values were obtained from available clinical records rather than study-mandated testing, and their availability was strongly structured by T2DM status. At least one fasting plasma glucose, post-prandial glucose, or HbA1c value was available for 643 of 690 participants with T2DM (93.2%), including 562 (81.4%) with all three measurements; none of the 414 participants without T2DM had any biochemical values available. Therefore, biochemical measures were not compared between groups and were not imputed. Among participants with T2DM with available measurements, the mean fasting plasma glucose level was 145.2 ± 43.3 mg/dL (*n* = 624), post-prandial glucose level was 236.7 ± 71.2 mg/dL (*n* = 584), and HbA1c level was 7.9 ± 1.3% (*n* = 619). Availability also varied by participating centre and geographic zone (zone comparison *p* < 0.001), and by selected participant and dietary characteristics within the T2DM group (Supplementary Table S3). Biochemical values are reported descriptively.

### 3.6. Descriptive Comparison with the Published STARCH 2014 Cohort

**Figure 3:**
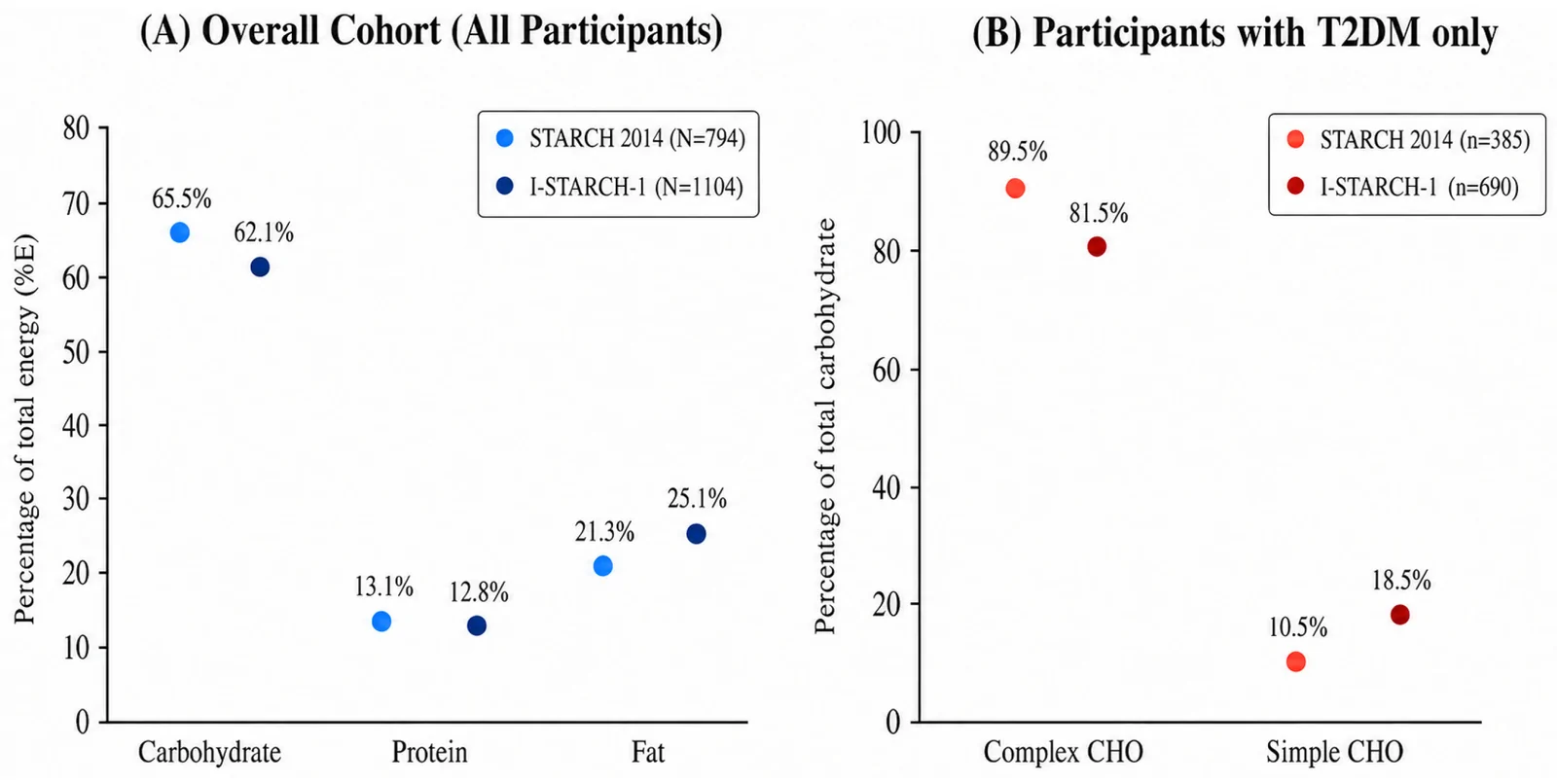
Descriptive comparison of macronutrient distribution and carbohydrate composition between the published STARCH 2014 cohort and I-STARCH-1. (**A**) Mean contributions of carbohydrate, protein and fat to total energy intake (%E) in the overall STARCH 2014 (*N* = 794) and I-STARCH-1 (*N* = 1104) cohorts. (**B**) Simple and complex carbohydrate expressed as percentages of total carbohydrate intake among participants with T2DM in STARCH 2014 (*n* = 385) and I-STARCH-1 (*n* = 690). Note: Points represent cohort means. No measures of dispersion are shown because the published dispersions for STARCH 2014 were wide and not comparable across cohorts, and no inferential testing was performed. STARCH 2014 values are published group means or, for the overall cohort, sample-size-weighted means of the published T2DM and non-T2DM groups [14]. Panel B is restricted to participants with T2DM because STARCH 2014 reported carbohydrate composition as a percentage of total carbohydrate only for that subgroup. The two studies were independent, non-harmonised cross-sectional surveys differing in eligibility criteria, dietary assessment and geographic framework; the differences shown are descriptive and do not constitute evidence of change over time. Dietary fibre was not reported in STARCH 2014 and is not shown. Abbreviations: CHO, carbohydrate; T2DM, type 2 diabetes mellitus; %E, percentage of total energy intake.

Published summary statistics from STARCH 2014 [14] are presented alongside the I-STARCH-1 values in Table 5 and Figure 3. In the overall cohort comparison and within both diabetes strata, the carbohydrate contribution was numerically lower and the fat contribution was numerically higher in the I-STARCH-1 group, whereas the protein contribution was similar. The largest numerical difference was in carbohydrate composition among participants with T2DM, where simple carbohydrate accounted for 10.5% of total carbohydrate in STARCH 2014 and 18.5% in I-STARCH-1. These comparisons are only contextual. The studies were independent, non-harmonised cross-sectional surveys with differences in eligibility criteria, dietary assessment, and geographic framework. STARCH 2014 recruited participants from 10 specialty centres across five regions, used a three-day dietary recall alongside an FFQ covering two typical working days and one weekend day, and required non-T2DM participants to not follow a diet plan or dietary advice. Dietary fibre was not reported in the STARCH 2014 study. No inferential testing was performed, and the observed differences should not be interpreted as evidence of changes over time.

### 3.7. Exploratory Analyses: Diet-Quality Index

The investigator-derived DQI was analysed descriptively because it has not been externally validated and has no established clinical thresholds. The mean participant-level DQI was 2.03 ± 6.09 overall, 3.78 ± 5.58 among participants with T2DM and −0.89 ± 5.78 among those without T2DM (Table 6). Higher values reflect greater protein and fibre relative to simple carbohydrates within the construct. An equivalent DQI could not be calculated for STARCH 2014 because dietary fibre was not reported; therefore, no historical comparison or inferential testing was performed for this dataset.

### 3.8. Exploratory Analyses: Protein–Fat Quality Index

The PFQI was analysed descriptively as an investigator-derived exploratory measure. The mean participant-level PFQI in I-STARCH-1 was 0.550 ± 0.248, with a median of 0.497 (IQR, 0.375–0.674) (Table 7). For comparison with STARCH 2014, for which participant-level data were unavailable, the ratio of cohort mean protein %E to cohort mean fat %E was calculated identically for both studies. This ratio was 0.615 in STARCH 2014 and 0.509 in I-STARCH-1, with numerically lower values in I-STARCH-1 in participants both with and without T2DM. The participant-level mean PFQI and ratio of cohort means are mathematically distinct and not interchangeable. No inferential testing was performed, and the historical comparison is subject to the non-harmonisation limitations described in Section 3.6.

## 4. Discussion

### 4.1. Principal Findings

In this multicentre clinic-based cohort of 1104 Indian adults, carbohydrates remained the predominant source of dietary energy, with roughly one-fifth of the total carbohydrate intake contributed by mono- and disaccharides, and fibre intake remained low. Participants with T2DM derived less energy from carbohydrates and more from protein and fat than those without T2DM and had a lower simple carbohydrate proportion; these differences persisted after adjustment for age and sex while accounting for centre clustering (Supplementary Table S2). The absolute fibre intake did not differ between the groups. Macronutrient composition was broadly similar across the four geographic zones, and the zone analyses were exploratory, unadjusted, and uncorrected for multiple comparisons. Because this study was cross-sectional, these differences describe the sampled groups and should not be interpreted causally.

### 4.2. Comparison with Previous Literature

The macronutrient profiles observed in this cohort closely resembled contemporary population-based Indian estimates. In the nationally representative ICMR-INDIAB survey-21, carbohydrates contributed 62.3%E, fat 25.2%E, and protein 12.0%E [5], compared with 62.1%E, 25.1%E, and 12.8%E, respectively, in the present study. This similarity does not establish the representativeness of the clinic-based convenience sample but indicates consistency with contemporary national estimates. Earlier Indian evidence likewise describes diets in which carbohydrate provides the majority of dietary energy, with substantial dependence on cereal-based foods and variation across regions and socioeconomic groups [7,26].

The same national survey also examined isocaloric macronutrient substitutions. Lower odds of newly diagnosed T2DM were observed when carbohydrate energy was replaced with selected protein sources, whereas substituting refined cereals with whole wheat or millet flour alone was not associated with a lower risk [5]. In the present cohort, mono- and disaccharides contributed approximately one-fifth of the total carbohydrate intake, alongside low fibre intake, supporting attention to carbohydrate composition as well as quantity. In the regional context, carbohydrate contribution exceeded 70%E in rural Bangladesh and was approximately 50.6%E in Chinese adults by 2015 [27,28]. Broader evidence from prospective cohorts likewise indicates that dietary patterns characterised by whole grains, legumes, fruits and vegetables are associated with lower risk of T2DM whereas patterns high in refined grains and added sugars are associated with higher risk [29], and comparison of Indian dietary intake with the EAT-Lancet reference diet has identified shortfalls in the same food groups [30]. These regional comparisons are therefore contextual.

### 4.3. Comparison with the STARCH 2014 Cohort

The comparison with STARCH 2014 should be interpreted as contextual rather than longitudinal because the two studies were independent, non-harmonised, cross-sectional cohorts that differed in sampling, eligibility criteria, dietary assessment, and geographic framework. Several explanations for the observed contrasts are plausible and cannot be disentangled from one another. National analyses have described increasing fat intake over time in India, while differences in dietary assessment methods may also contribute to variation in estimated macronutrient intake [1,31,32]. The cohort composition also differed, with STARCH 2014 excluding individuals following a dietary plan from its non-diabetes group, whereas the present cohort included participants reporting dietary restriction. In addition, STARCH 2014 did not report dietary fibre, precluding reconstruction of an equivalent historical DQI, and published dispersion estimates in the earlier cohort were very wide, precluding meaningful inferential comparison. Therefore, the PFQI comparison was restricted to a like-for-like ratio of cohort means and interpreted descriptively. None of these historical contrasts should be considered as evidence of change over time.

### 4.4. Interpretation of Simple and Complex Carbohydrate

In this study, carbohydrate quality was characterised compositionally through the balance of mono- and disaccharides to polysaccharides and the density of dietary fibre, two dimensions consistently associated with cardiometabolic outcomes [2]. Both dimensions point the same way in this cohort: mono- and disaccharides contributed approximately one-fifth of the total carbohydrate, while fibre density remained low, a combination that suggests scope for improvement independent of total carbohydrate intake. Compositional framing also sets the boundary of what can be inferred. Mono- and disaccharides include intrinsic sugars from fruit and milk alongside added sugars, and the polysaccharide fraction spans intact wholegrain foods and refined starches that differ appreciably in food matrix and glycaemic behaviour [9,33]. This heterogeneity is consequential rather than technical: national evidence indicates that substituting whole wheat or millet flour for refined cereals was not associated with lower metabolic risk, and that glycaemic characteristics vary widely across Indian carbohydrate foods [3,5]. Therefore, carbohydrate composition and fibre density are best read together as indicators of where dietary improvement is plausible rather than as substitutes for the direct measurement of free sugars or glycaemic response.

### 4.5. Interpretation of Differences Between Participants With and Without T2DM

Participants with T2DM derived less energy from carbohydrate and more from protein and fat, and had a lower simple-carbohydrate proportion, than those without T2DM; these differences persisted after adjustment for age, sex and centre clustering, and after further adjustment for restricted-diet status, diet type and exercise (Supplementary Table S2). The absolute fibre intake did not differ. One possible explanation is post-diagnosis dietary modification following counselling or greater awareness of recommendations [34,35], consistent with the higher proportion of participants with T2DM reporting dietary restriction (55.7% vs. 43.0%) and their lower total energy intake. Reporting bias may also contribute, as awareness of dietary recommendations is greater among those with diagnosed diabetes [36,37]. Detailed medication regimens, disease duration and comorbidities were not available for analysis. Because the design is cross-sectional, the temporal ordering of diagnosis and intake cannot be established, and these differences characterise the sampled groups rather than any effect of diabetes status on diet.

### 4.6. Geographic Variation

Macronutrient and carbohydrate composition were broadly similar across the four geographic zones, with carbohydrates remaining the predominant energy source throughout. Overall zone comparisons were statistically significant for carbohydrate, protein and fat contribution, dietary fibre and carbohydrate composition. The largest between-zone spreads were 1.6 g/day for fibre intake and 3.7 percentage points of energy for fat contribution. These analyses were exploratory and unadjusted, with no correction for multiple comparisons and no pairwise contrasts, so the overall *p* values should not be interpreted as identifying specific differences between zones. Because participants were recruited by convenience sampling from diabetes-focused centres, the findings describe heterogeneity within the participating clinic-based sample and should not be generalised as evidence of either regional similarity or regional differences across India.

### 4.7. Clinical and Public Health Implications

These findings support the importance of balanced macronutrient intake, fibre adequacy, and carbohydrate composition rather than carbohydrate quantity alone. The mean protein intake was 12.8%E, below the 15–20%E range recommended in contemporary Indian guidance for T2DM, although individual protein adequacy cannot be inferred from percentage energy intake alone and should be considered in relation to age, renal function, and clinical context [11,38]. National dietary data indicate that cereals and millets constitute the largest single contributor to protein intake among Indian adults; therefore, both protein quantity and source quality warrant consideration [39,40]. This may be relevant during intentional weight loss, including with GLP-1-based pharmacotherapy, where preservation of lean mass is a clinical consideration, and adequate protein intake together with resistance exercise may be beneficial [41,42]. Adequate dietary fibre intake may be of particular relevance in older adults, in whom fibre intake has been linked to inflammatory and metabolic markers [43]. In older adults, obesity may mask malnutrition, supporting the assessment of nutritional status beyond body size alone; participants in the present cohort were aged up to 88 years, but nutritional status was not assessed, so no inference regarding malnutrition can be made [44]. Because free and added sugars and specific food sources were not separately quantified, these data do not support recommendations specifically targeting free sugar intake. Rather, the findings provide descriptive support for existing Indian guidance promoting balanced diets and fibre-rich foods, including whole grains, pulses, vegetables and fruits, while dietary recommendations should remain individualised [8,10].

### 4.8. Strengths and Limitations

The main strengths are a large contemporary sample of 1104 adults with and without T2DM recruited from 29 centres across 14 states, standardised procedures at all sites, a common dietary method permitting direct between-group comparison, and centre identifiers enabling mixed-effects models that accounted for clustering. Overall, the macronutrient distribution closely matched contemporary national estimates.

The cross-sectional design precludes causal inference, and convenience sampling from diabetes-focused centres limits generalisability beyond healthcare-seeking adults; recruitment was deliberately enriched for T2DM, so the observed proportion does not reflect prevalence. Such recruitment may also have overrepresented individuals with greater health awareness or access to dietary advice, potentially biasing estimates towards more favourable patterns by an amount that cannot be quantified here. Dietary intake was self-reported and subject to recall and under-reporting bias, and calendar dates were not captured, so recall-day spacing and weekday or weekend distribution could not be verified. The implementation-level similarity function and the provenance of food-specific gram-equivalent values were not retained, limiting full computational reproducibility, although all food-code assignments were human-confirmed and a subset of portion conversions independently cross-checked. Waist circumference was estimated rather than measured and used descriptively only, and simple carbohydrates represented total mono- and disaccharides without separation of intrinsic, added, and free sugars. As no study-mandated glycaemic testing was performed, undiagnosed diabetes in the non-T2DM group cannot be excluded and could have attenuated the observed between-group differences. Biochemical values were entirely absent in that group, precluding between-group comparison or defensible imputation, and residual confounding from medication use, disease duration, and socioeconomic status remains possible. Geographic-zone and other exploratory analyses were unadjusted and uncorrected for multiple comparisons, and the DQI and PFQI were investigator-derived and unvalidated. Comparisons with STARCH 2014 rest on non-harmonised summary statistics; dietary fibre was not reported in that study, precluding an equivalent historical DQI, and no inferential testing of historical contrasts was performed.

### 4.9. Future Research

Population-based sampling and repeated or prospective dietary assessments would help determine whether the patterns observed in this clinic-based cohort reflect broader population intake and how they vary over time. Seasonally repeated recalls could additionally capture within-year variation not assessed here. Comparisons with earlier Indian cohorts would be strengthened by harmonised individual-level data and a common dietary assessment method, enabling inferential comparisons that were not possible in the present historical analysis. Direct anthropometric measurements and biochemical assessments under a common protocol in participants with and without T2DM would address important measurement and availability limitations. Future dietary assessments should distinguish between intrinsic, added, and free sugars and more explicitly characterise food sources, processing, and glycaemic properties. Finally, the investigator-derived DQI and PFQI require external validation against established dietary quality measures and clinically relevant outcomes before broader research, clinical, or policy use.

## 5. Conclusions

In this multicentre clinic-based cross-sectional study of 1104 Indian adults with and without T2DM, carbohydrates remained the predominant source of dietary energy, fibre intake was low, and mono- and disaccharides accounted for approximately one-fifth of the total carbohydrate intake. Participants with T2DM derived less energy from carbohydrates and more from protein and fat than those without T2DM, and these differences persisted after adjustment for age and sex while accounting for clustering within centres. Absolute fibre intake did not differ between groups. Macronutrient distribution and carbohydrate compositions showed only modest variation across the four geographic zones. Descriptive comparison with STARCH 2014 showed differences in macronutrient composition, but the cohorts were non-harmonised and these contrasts should not be interpreted as evidence of change over time. The findings describe healthcare-seeking adults attending diabetes-focused centres rather than the general Indian population and support attention to macronutrient balance, fibre adequacy and carbohydrate composition alongside carbohydrate quantity. Population-based and longitudinal studies are needed to determine whether these patterns extend beyond the present setting.

## Figures and Tables

**Table 1:** Eligibility criteria for study participants.

Category	Criteria
Inclusion, General	Adults aged ≥ 18 years; provided written informed consent; relevant clinical documentation available from the preceding 3 months; clinically stable for ≥30 days.
Inclusion, T2DM	Confirmed diagnosis of T2DM according to American Diabetes Association (ADA) criteria, with a diagnosis of ≥12 months’ duration; stable glucose-lowering medication for ≥3 months; no hospitalisation within the preceding 3 months.
Inclusion, Non-T2DM	No known or documented history of diabetes mellitus; not receiving glucose-lowering medication; not following a medically prescribed restricted diet at screening; fasting plasma glucose < 126 mg/dL or HbA1c < 6.5% where such values were available.
Exclusion, General	Pregnancy or lactation; severe cognitive impairment; active malignancy or terminal illness; hospitalisation within the preceding 30 days; incomplete or unreliable dietary data.
Exclusion, Medical	Type 1 diabetes or secondary diabetes; chronic kidney disease (CKD; eGFR < 30 mL/min/1.73 m^2^); active liver disease or cirrhosis; eating disorders.
Exclusion, Lifestyle	Participation in structured weight-loss programmes; major dietary change within the preceding 3 months; inability to complete dietary recalls.

Note: Age eligibility was ≥18 years with no upper limit; the observed range was 19 to 88 years. Clinical documentation from the preceding three months was required for eligibility but did not necessarily include recent biochemical values. The published protocol specified a T2DM diagnosis of ≥12 months’ duration; however, diagnosis duration was not retained in the final analytical dataset and could not be analysed. The restricted-diet variable in the analytic dataset records any self-reported dietary restriction and is broader than the medically prescribed dietary restriction referred to at screening; it was used as a descriptive covariate. Abbreviations: ADA, American Diabetes Association; CKD, chronic kidney disease; eGFR, estimated glomerular filtration rate; HbA1c, glycated haemoglobin; T2DM, type 2 diabetes mellitus.

**Table 2:** Baseline demographic and lifestyle characteristics of the study participants (*N* = 1104).

Characteristic	Overall (*N* = 1104)	T2DM (*n* = 690)	Non-T2DM (*n* = 414)	*p* Value
Age (years), mean ± SD	49.8 ± 11.9	51.3 ± 11.2	47.3 ± 12.6	<0.001
**Sex, *n* (%)**				0.504
Male	541 (49.0)	344 (49.9)	197 (47.6)	
Female	563 (51.0)	346 (50.1)	217 (52.4)	
BMI (kg/m^2^), mean ± SD	24.3 ± 3.6	24.4 ± 3.6	24.1 ± 3.6	0.183
Estimated waist circumference (cm), mean ± SD	92.2 ± 10.6	93.0 ± 10.3	90.8 ± 10.8	<0.001
**Diet type, *n* (%)**				0.368
Vegetarian	339 (30.7)	210 (30.4)	129 (31.2)	
Eggetarian	64 (5.8)	35 (5.1)	29 (7.0)	
Non-vegetarian	701 (63.5)	445 (64.5)	256 (61.8)	
Restricted diet during the past year, n (%)	562 (50.9)	384 (55.7)	178 (43.0)	<0.001
Exercise participation, n (%)	498 (45.1)	318 (46.1)	180 (43.5)	0.435
Exercise duration (min/week), mean ± SD	54.7 ± 76.1	55.6 ± 76.2	53.0 ± 76.0	0.579
Alcohol consumption, n (%)	184 (16.7)	113 (16.4)	71 (17.1)	0.802
Outside or delivered meals (per week), mean ± SD	1.6 ± 1.3	1.6 ± 1.3	1.7 ± 1.4	0.253
Daily meal frequency, mean ± SD	3.5 ± 1.0	3.5 ± 0.9	3.6 ± 1.0	0.169
Daily water intake (L/day), mean ± SD	2.1 ± 0.8	2.1 ± 0.8	2.2 ± 0.9	0.320
**Geographic zone, *n* (%)**				0.055
North	220 (19.9)	140 (20.3)	80 (19.3)	
South	328 (29.7)	223 (32.3)	105 (25.4)	
East	343 (31.1)	203 (29.4)	140 (33.8)	
West	213 (19.3)	124 (18.0)	89 (21.5)	

Note: Data are presented as mean ± SD or *n* (%). *p* Values were derived using independent-samples *t*-tests for continuous variables and chi-square tests for categorical variables. Restricted diet included any self-reported dietary restriction during the preceding year. Estimated waist circumference was reported descriptively. Categorical percentages are column percentages. T2DM duration was not captured. Abbreviations: BMI, body mass index; SD, standard deviation; T2DM, type 2 diabetes mellitus.

**Table 3:** Energy and macronutrient profile by T2DM status.

Variable	Overall (*N* = 1104)	T2DM (*n* = 690)	Non-T2DM (*n* = 414)	*p* Value
Energy (kcal/day)	1778.6 ± 369.9	1616.0 ± 280.4	2049.6 ± 341.4	<0.001
Carbohydrate (%E)	62.1 ± 5.4	61.0 ± 5.4	64.1 ± 5.0	<0.001
Protein (%E)	12.8 ± 3.5	13.1 ± 3.6	12.2 ± 3.3	<0.001
Fat (%E)	25.1 ± 5.5	25.9 ± 5.5	23.7 ± 5.3	<0.001
Dietary fibre (g/day)	15.4 ± 3.2	15.5 ± 3.2	15.2 ± 3.2	0.071
Dietary fibre (g/1000 kcal)	9.1 ± 2.8	9.9 ± 2.9	7.6 ± 2.1	<0.001
Simple carbohydrate (% of total carbohydrate)	20.1 ± 6.5	18.5 ± 5.8	22.8 ± 6.6	<0.001
Complex carbohydrate (% of total carbohydrate)	79.9 ± 6.5	81.5 ± 5.8	77.2 ± 6.6	<0.001

Note: Values are mean ± SD. *p* Values are from independent-samples *t*-tests and represent unadjusted between-group comparisons. Simple and complex carbohydrate are expressed as percentages of total carbohydrate intake and sum to 100%. Adjusted comparisons of the five dietary outcomes are reported in Supplementary Table S2. Abbreviations: %E, percentage of total energy intake; SD, standard deviation; T2DM, type 2 diabetes mellitus.

**Table 4:** Geographic-zone distribution of energy, macronutrient intake and carbohydrate composition.

Geographic Zone	*n*	Energy (kcal/day)	Carbohydrate (%E)	Protein (%E)	Fat (%E)	Fibre (g/day)	Simple CHO (% of Total CHO)	Complex CHO (% of Total CHO)
North	220	1783 ± 368	61.5 ± 5.5	11.9 ± 3.6	26.6 ± 5.3	15.3 ± 3.2	19.3 ± 6.1	80.7 ± 6.1
South	328	1792 ± 361	61.5 ± 5.1	12.3 ± 3.4	26.2 ± 4.9	14.8 ± 2.8	21.3 ± 6.9	78.7 ± 6.9
East	343	1775 ± 393	63.2 ± 5.2	13.9 ± 3.0	22.9 ± 5.4	15.4 ± 3.0	19.5 ± 5.9	80.6 ± 5.9
West	213	1759 ± 349	62.1 ± 5.9	12.6 ± 4.0	25.3 ± 5.6	16.4 ± 3.6	20.1 ± 6.8	79.9 ± 6.8
*p* value		0.777	<0.001	<0.001	<0.001	<0.001	<0.001	<0.001

Note: Values are mean ± SD. *p* Values are from one-way ANOVA comparing the four geographic zones. Simple and complex carbohydrate were derived from continuous participant-level values and sum to 100%; displayed group means may differ marginally from 100% because of rounding. Zone comparisons were exploratory and unadjusted; no correction for multiple comparisons was applied, and no pairwise between-zone contrasts were tested.

**Table 5:** Descriptive comparison between the published STARCH 2014 and I-STARCH-1 cohorts.

Variable	STARCH 2014	I-STARCH-1	Difference, I-STARCH-1 Minus STARCH 2014
**Overall cohort**	***N* = 794**	***N* = 1104**	
Energy (kcal/day)	1848.6	1778.6	−70.1
Carbohydrate (%E)	65.5	62.1	−3.4
Protein (%E)	13.1	12.8	−0.4
Fat (%E)	21.3	25.1	+3.7
**Participants with T2DM**	***n* = 385**	***n* = 690**	
Energy (kcal/day)	1547.5	1616.0	+68.5
Carbohydrate (%E)	64.1	61.0	−3.1
Protein (%E)	14.3	13.1	−1.2
Fat (%E)	21.6	25.9	+4.3
Simple carbohydrate (% of total CHO)	10.5	18.5	+8.0
Complex carbohydrate (% of total CHO)	89.5	81.5	−8.0
**Participants without T2DM**	***n* = 409**	***n* = 414**	
Energy (kcal/day)	2132.0	2049.6	−82.4
Carbohydrate (%E)	66.8	64.1	−2.7
Protein (%E)	12.0	12.2	+0.2
Fat (%E)	21.1	23.7	+2.6

Note: STARCH 2014 values are published group means or, for the overall cohort, sample-size-weighted means of the published T2DM and non-T2DM groups. The published dietary summaries comprised 385 participants with T2DM and 409 without T2DM. STARCH 2014 directly reported complex carbohydrate as 89.5% of total carbohydrate among participants with T2DM; simple carbohydrate was therefore derived as 10.5%. Equivalent carbohydrate-composition measures were unavailable for the overall and non-T2DM STARCH cohorts. Dietary fibre was not reported. Differences were calculated from unrounded values and may therefore differ by 0.1 from differences between the rounded values shown. Comparisons are descriptive only; no *p* values or confidence intervals were calculated. Abbreviations: CHO, carbohydrate; T2DM, type 2 diabetes mellitus; %E, percentage of total energy intake.

**Table 6:** Exploratory Diet-Quality Index in the I-STARCH-1 cohort.

Group	*n*	DQI, Mean ± SD	Median (IQR)	Range
Overall	1104	2.03 ± 6.09	2.02 (−1.78 to 5.95)	−17.11 to 20.88
Participants with T2DM	690	3.78 ± 5.58	3.63 (−0.15 to 7.60)	−13.37 to 20.88
Participants without T2DM	414	−0.89 ± 5.78	−0.55 (−4.95 to 3.28)	−17.11 to 16.93

Note: DQI = protein (%E) + fibre (%E) − simple carbohydrate (%E), calculated at participant level. All three DQI components used the same fibre-inclusive energy denominator, with fibre assigned 2 kcal/g, as described in Section 2.9. The DQI is investigator-derived, has not been externally validated and has no established clinical thresholds. STARCH 2014 did not report dietary fibre, so an equivalent historical DQI could not be reconstructed. No historical or between-group inferential testing of the DQI was performed. Abbreviations: DQI, Diet-Quality Index; IQR, interquartile range; SD, standard deviation; T2DM, type 2 diabetes mellitus; %E, percentage of total energy intake.

**Table 7:** Exploratory Protein–Fat Quality Index. (**A**) I-STARCH-1 participant-level distribution. (**B**) Descriptive comparison with STARCH 2014 using ratios of cohort means.

(**A**)			
**Group**	***n***	**Mean ± SD**	**Median (IQR)**
Overall	1104	0.550 ± 0.248	0.497 (0.375 to 0.674)
Participants with T2DM	690	0.545 ± 0.239	0.478 (0.368 to 0.681)
Participants without T2DM	414	0.558 ± 0.261	0.521 (0.398 to 0.667)
(**B**)			
**Group**	**STARCH 2014**	**I-STARCH-1**	**Difference**
Overall	0.615	0.509	−0.106
Participants with T2DM	0.665	0.507	−0.158
Participants without T2DM	0.568	0.513	−0.055

Note: PFQI = protein (%E) divided by fat (%E). (**A**) reports participant-level ratios calculated within I-STARCH-1. (**B**) reports the ratio of cohort mean protein %E to cohort mean fat %E, calculated identically for both studies because participant-level data were unavailable for STARCH 2014. These quantities are mathematically distinct and should not be compared interchangeably. STARCH 2014 values were derived from published group means [14], with the overall value based on sample-size-weighted group means. Differences are arithmetic and descriptive only; no *p* values or confidence intervals were calculated. PFQI has not been externally validated and has no established clinical thresholds. Abbreviations: IQR, interquartile range; PFQI, Protein–Fat Quality Index; SD, standard deviation; T2DM, type 2 diabetes mellitus; %E, percentage of total energy intake.

## Data Availability

The data presented in this study are available upon request from the corresponding author. They are not publicly available because they contain information that could compromise participant privacy and because participant consent was not provided for open data deposition. The completed STROBE-nut checklist, dietary survey questionnaire, and data-cleaning audit trail are provided in the Supplementary Materials. The de-identified analytic dataset and participating-centre enrolment details are available from the corresponding author on reasonable request, subject to ethics approval, institutional permission and an appropriate data-use agreement.

## Supplementary Materials

The following supporting information can be downloaded at: https://www.mdpi.com/article/10.3390/nu18182993/s1, Table S1: STROBE-nut Checklist for the I-STARCH-1 Study; Table S2: Unadjusted and Centre-Adjusted Differences in Primary Dietary Outcomes by T2DM Status; Table S3: Biochemical Data Availability and Characteristics According to Availability; File S1: Dietary Survey Questionnaire; File S2: Data-Cleaning Audit Trail; Figure S1: Macronutrient distribution and carbohydrate composition according to diabetes status; Figure S2: Geographic distribution of macronutrient intake across the I-STARCH-1 cohort.
