# Supplementary material for: Macronutrient Intake and Carbohydrate Quality in Indian Adults With and Without Type 2 Diabetes: The Multicentre I-STARCH-1 Cross-Sectional Study
Source: Nutrients. 2026 Sep 13;18(18):2993. doi: 10.3390/nu18182993 (PMC13610836; doi:10.3390/nu18182993)
Supplement: Supplementary file 1 [file nutrients-18-02993-s001.zip › nutrients-4525144-supplementary.pdf]

Supplementary Materials

Macronutrient Intake and Carbohydrate Quality in Indian Adults With and Without Type 2 Diabetes: The Multicentre I-STARCH-1 Cross-Sectional Study

Kapoor N, Kalra S, Deshpande N, Salis SS, Gadia S, Anthuvan T

Contents

Supplementary Table S1. STROBE-nut Checklist for the I-STARCH-1 Study

Supplementary Table S2. Unadjusted and Centre-Adjusted Differences in Primary Dietary Outcomes by T2DM Status

Supplementary Table S3. Biochemical Data Availability and Characteristics According to Availability

Supplementary File S1. Dietary Survey Questionnaire

Supplementary File S2. Data-Cleaning Audit Trail

Supplementary Figure S1. Macronutrient distribution and carbohydrate composition according to diabetes status.

Supplementary Figure S2. Geographic distribution of macronutrient intake across the I-STARCH-1 cohort.

Supplementary Table S1. STROBE-nut Checklist for the I-STARCH-1 Study

Adapted from: Lachat C, Hawwash D, Ocké MC, Berg C, Forsum E, Hörnell A, et al. Strengthening the Reporting of Observational Studies in Epidemiology-Nutritional Epidemiology (STROBE-nut). PLOS Med. 2016;13(6):e1002036.

Item	Item No.	STROBE Recommendation	Extension for Nutritional Epidemiology (STROBE-nut)	Reported in
Title and abstract	1	(a) Indicate the study's design with a commonly used term in the title or the abstract. (b) Provide in the abstract an informative and balanced summary of what was done and what was found.	nut-1 State the dietary/nutritional assessment method(s) used in the title, abstract, or keywords.	Title - described as a multicentre cross-sectional study. Abstract - summarises objectives, design, methods and key results; states that dietary intake was assessed using a structured three-day dietary recall coded against the Indian Nutrient Databank (INDB).
Background / rationale	2	Explain the scientific background and rationale for the investigation being reported.	-	Section 1 (Introduction).
Objectives	3	State specific objectives, including any pre-specified hypotheses.	-	Section 1 (Introduction), final paragraph - primary, secondary and exploratory objectives and the study hypothesis are stated. The hypothesis is non-directional.
Study design	4	Present key elements of study design early in the paper.	-	Section 2.1 - multicentre, cross-sectional observational study.

Setting	5	Describe the setting, locations, and relevant dates, including periods of recruitment, exposure, follow-up, and data collection.	nut-5 Describe any characteristics of the study settings that might affect the dietary intake or nutritional status of the participants, if applicable.	Section 2.1 - 29 diabetes-focused healthcare centres across 14 Indian states, spanning the north, south, east and west geographic zones; conducted in 2025. Centres were led by practising diabetologists or physicians (62.1%), endocrinologists (34.5%) and a dietitian (3.4%). Community and other non-clinical settings contributed no participants to the final analysed cohort. Standardised protocols applied under central coordination. Section 2.5 - recruitment and participant flow.
Participant s	6	(a) Cross-sectional study - Give the eligibility criteria, and the sources and methods of selection of participants. (b) For matched studies, give matching criteria and number of exposed and unexposed.	nut-6 Report particular dietary, physiological, or nutritional characteristics that were considered when selecting the target population.	Sections 2.3 and 2.4; Table 1 - adults aged ≥18 years, with or without T2DM; no upper age limit specified; observed range 19–88 years (mean 49.8 ± 11.9). Eligibility required clinical documentation from the preceding three months, which did not necessarily include recent biochemical values. Full criteria in Table 1. The published protocol specified a T2DM diagnosis of ≥12 months' duration; diagnosis duration was not retained in the analytic dataset and could not be verified. Recruitment used convenience sampling and was prospectively enriched for T2DM. (b) Not applicable - not a matched study.

Variables	7	Clearly define all outcomes, exposures, predictors, potential confounders, and effect modifiers. Give diagnostic criteria, if applicable.	nut-7.1 Clearly define foods, food groups, nutrients, or other food components. nut-7.2 When using dietary patterns or indices, describe the methods to obtain them and their nutritional properties.	Sections 2.9–2.12 - outcomes defined as carbohydrate, protein and fat intake as percentage of total energy (%E); simple and complex carbohydrate as percentage of total carbohydrate; dietary fibre as g/day and g/1,000 kcal. Simple carbohydrate defined as mono- and disaccharides at food-code level; intrinsic, added and free sugars could not be distinguished. Section 2.12 - Diet-Quality Index (DQI = protein %E + fibre %E − simple carbohydrate %E) and Protein–Fat Quality Index (PFQI = protein %E ÷ fat %E) defined; both investigator-derived, not externally validated, reported as exploratory. Section 2.4 - T2DM ascertainment hierarchy.

Data sources / measurement	8	For each variable of interest, give sources of data and details of methods of assessment (measurement). Describe comparability of assessment methods if there is more than one group.	nut-8.1 Describe the dietary assessment method(s) - portion size estimation, number of days and items recorded, how it was developed and administered, and how quality was assured. Report if and how supplement intake was assessed. nut-8.2 Describe and justify food composition data used; explain matching procedure and conversion factors. nut-8.3 Describe nutrient requirements, recommendations or dietary guidelines used, if applicable. nut-8.4 When using nutritional biomarkers, additionally use the STROBE-ME extension. nut-8.5 Describe assessment of non-dietary data and timing relative to dietary assessment. nut-8.6 Report on the validity of the dietary or nutritional assessment methods and any internal or external validation used, if applicable.	nut-8.1 - Section 2.7: structured three-day dietary recall administered face-to-face by trained investigators using a standardised multi-pass technique and household measures; intake recorded separately for Day 1, Day 2 and Day 3. Calendar dates and day of week were not captured, so recall-day spacing and weekday/weekend distribution could not be verified. Questionnaire provided in Supplementary File S1. Field supervisors reviewed recalls and conducted random verification calls. Dietary supplement intake was not separately assessed. nut-8.2 - Section 2.8: food descriptions standardised and matched by a custom fuzzy-string-matching computational tool against the Indian Nutrient Databank (INDB), whose composition data derive primarily from ICMR–NIN IFCT 2017; candidate matches confirmed by investigator or dietitian review before final coding; mixed dishes decomposed to ingredient level; regional, ambiguous and branded items resolved by human review. nut-8.3 - Section 4.7: intakes discussed against national dietary recommendations; no formal adequacy scoring performed. nut-8.4 - Not applicable; no biomarkers of intake were measured.

Bias	9	Describe any efforts to address potential sources of bias.	nut-9 Report how bias in dietary or nutritional assessment was addressed - e.g., misreporting, changes in habits as a result of being measured, or data imputation from other sources.	Sections 2.5 and 2.13 - standardised quality checks; energy plausibility range 800–4,000 kcal/day applied at data review; investigator training and standardised procedures across centres. A source-verification audit identified and corrected three transcription errors in total energy and rescaled 27 HbA1c values recorded as proportions; the audit trail is provided in Supplementary File S2. Section 4.8 - recall bias, social desirability bias and under-reporting acknowledged. Structural absence of biochemical data in the non-T2DM group is reported in Section 2.13 and Supplementary Table S3.
Study size	10	Explain how the study size was arrived at.	-	Section 2.6 - no formal a priori sample size or power calculation was conducted; a pragmatic recruitment target of approximately 40 participants per centre was applied. Achieved precision for the primary outcome is reported.
Quantitative variables	11	Explain how quantitative variables were handled in the analyses. If applicable, describe which groupings were chosen and why.	nut-11 Explain categorisation of dietary/nutritional data (e.g., N-tiles, handling of non-consumers) and the choice of reference category, if applicable.	Section 2.14 - continuous variables presented as mean ± SD or median (IQR) according to distribution; categorical variables as frequencies and percentages. No N-tile categorisation, non-consumer grouping or reference category applied; dietary variables analysed as continuous measures averaged across the three recall days.

Statistical methods	12	(a) Describe all statistical methods, including those used to control for confounding. (b) Describe any methods used to examine subgroups and interactions. (c) Explain how missing data were addressed. (d) Cross-sectional study - describe analytical methods taking account of sampling strategy, if applicable. (e) Describe any sensitivity analyses.	nut-12.1 Describe any statistical method used to combine dietary or nutritional data, if applicable. nut-12.2 Describe and justify the method for energy adjustments, intake modelling, and use of weighting factors, if applicable. nut-12.3 Report any adjustments for measurement error, e.g., from a validity or calibration study.	(a) Section 2.14 - Welch two-sample t-tests or one-way ANOVA for continuous variables; Mann–Whitney U or Kruskal–Wallis for non-normally distributed variables; chi-square for categorical variables. Confounder-adjusted estimates obtained from linear mixed-effects models with participating centre as a random intercept, fitted by restricted maximum likelihood; the primary model included T2DM status, age and sex as fixed effects, and a sensitivity model additionally included restricted-diet status, diet type and exercise status. Intraclass correlation coefficients reported. IBM SPSS Statistics v29.0 and Python 3.12 (statsmodels 0.15.0); two-sided $P < 0.05$. (b) Sections 3.3–3.4 - subgroup comparisons by T2DM status and geographic zone; no interaction modelling. (c) Section 2.13 - no imputation. Primary dietary outcomes were complete; biochemical variables were entirely absent in the non-T2DM group, rendering imputation inapplicable. (d) Section 2.1 - convenience sampling; centre clustering addressed through random intercepts; no sampling weights applied. (e) Sensitivity model reported in Supplementary Table S2. nut-12.1 - Section 2.9: nutrient values averaged across the three recall days.

Participant s	13	(a) Report the numbers of individuals at each stage of the study. (b) Give reasons for non-participation at each stage. (c) Consider use of a flow diagram.	nut-13 Report the number of individuals excluded based on missing, incomplete, or implausible dietary/nutritional data.	Section 2.5 and Figure 1 - 1,358 individuals screened; 1,137 (83.7%) eligible and enrolled; 221 excluded for ineligibility, declined participation or incomplete baseline information. A further 33 participants were excluded after data quality review owing to incomplete or unreliable dietary recalls, implausible energy intake, or recent participation in specialised diet programmemes (disaggregated counts not recorded). Final analytic sample 1,104 (690 with T2DM; 414 without). Participant flow shown in Figure 1. The number approached before formal screening was not consistently recorded; a conventional response rate could not be calculated.
Descriptive data	14	(a) Give characteristics of study participants (demographic, clinical, social) and information on exposures and potential confounders. (b) Indicate the number of participants with missing data for each variable of interest.	nut-14 Give the distribution of participant characteristics across the exposure variables, if applicable. Specify if food consumption of the total population or consumers only was used to obtain results.	Section 3.1 and Table 2 - age, sex, BMI, estimated waist circumference, diet type, restricted-diet status, exercise participation and duration, alcohol use, outside meals, meal frequency, water intake and geographic zone, presented overall and by T2DM status. Section 2.13 - missingness for biochemical covariates following the coding audit (fasting plasma glucose 43.5%, post-prandial glucose 47.1%, HbA1c 43.9%); primary dietary outcomes complete for all 1,104 participants. Supplementary Table S3 - availability of biochemical data by T2DM status. Results are based on the total analytic population, not consumers only.

Outcome data	15	Cross-sectional study - Report numbers of outcome events or summary measures.	-	Sections 3.2–3.4; Tables 3 and 4 - summary measures for energy, macronutrient intake, carbohydrate composition and dietary fibre, reported overall, by T2DM status and by geographic zone. Section 3.5 and Supplementary Table S3 - descriptive biochemical findings among participants with T2DM.
Main results	16	(a) Give unadjusted estimates and, if applicable, confounder-adjusted estimates and their precision (e.g., 95% CI). Make clear which confounders were adjusted for and why. (b) Report category boundaries when continuous variables were categorised. (c) If relevant, consider translating relative-risk estimates into absolute risk.	nut-16 Specify if nutrient intakes are reported with or without inclusion of dietary supplement intake, if applicable.	(a) Sections 3.2–3.4; Tables 3 and 4 - unadjusted estimates. Section 3.3 and Supplementary Table S2 - confounder-adjusted mean differences with 95% confidence intervals from mixed-effects models adjusted for age and sex with centre as a random intercept; a sensitivity model additionally adjusted for restricted-diet status, diet type and exercise status. Adjustment variables were selected as measured characteristics differing between groups; behavioural covariates were placed in the sensitivity model because they may be influenced by diagnosis. Sections 3.6–3.8; Tables 5–7 - historical and exploratory comparisons are descriptive; no P values or confidence intervals are reported for these contrasts. (b) Not applicable - continuous variables were not categorised. (c) Not applicable - no relative-risk estimates. nut-16 - Nutrient intakes reflect food and beverage sources only; supplements not included.

Other analyses	17	Report other analyses done - e.g., subgroup, interaction, and sensitivity analyses.	nut-17 Report any sensitivity analysis (e.g., exclusion of misreporters or outliers) and data imputation, if applicable.	Sections 3.3–3.5 - subgroup comparisons by T2DM status and geographic zone; characterisation of biochemical-data availability (Supplementary Table S3). Section 3.6 - descriptive comparison with the published STARCH 2014 cohort, without inferential testing. Sections 3.7–3.8 - exploratory DQI and PFQI analyses, explicitly identified as exploratory. Supplementary Table S2 - sensitivity model with additional behavioural covariates. Records with implausible energy intake were corrected against source records rather than excluded (Section 2.13).
Key results	18	Summarise key results with reference to study objectives.	-	Section 4.1 (Principal Findings) and Section 5 (Conclusions).
Limitations	19	Discuss limitations of the study, taking into account sources of potential bias or imprecision. Discuss both direction and magnitude of any potential bias.	nut-19 Describe the main limitations of the data sources and assessment methods used and implications for interpretation of the findings.	Section 4.8 - cross-sectional design precluding causal inference and temporal ordering; convenience clinic-based recruitment and deliberate T2DM enrichment limiting generalisability; self-reported recalls subject to recall and under-reporting bias; unverified recall-day spacing and weekday/weekend distribution; estimated rather than measured waist circumference; inability to separate intrinsic, added and free sugars; possible undiagnosed diabetes in the non-T2DM group, with the direction of any resulting bias stated as attenuating; complete absence of biochemical data in the non-T2DM group; residual confounding; absence of multiple-comparison correction for secondary and exploratory analyses; unvalidated DQI and PFQI; non-harmonised STARCH 2014 comparison and the impossibility of

				reconstructing a historical DQI because fibre was not reported.
Interpretation	20	Give a cautious overall interpretation of results considering objectives, limitations, multiplicity of analyses, results from similar studies, and other relevant evidence.	nut-20 Report the nutritional relevance of the findings, given the complexity of diet or nutrition as an exposure.	Sections 4.2–4.7 - findings interpreted against contemporary Indian national evidence and regional comparators; interpretation of carbohydrate composition; interpretation of differences by diabetes status; geographic variation; clinical and public-health implications. Comparisons with STARCH 2014 are presented as descriptive contrasts between independent, non-harmonised cross-sectional cohorts and explicitly not as evidence of temporal change.

Generalisability	21	Discuss the generalisability (external validity) of the study results.	-	Sections 2.1, 4.6 and 4.8 - convenience sampling across 29 diabetes-focused centres in 14 states; the sample is geographically diverse but not population-representative. Findings are framed as describing healthcare-seeking adults attending diabetes-focused centres. Observed similarity across zones is not interpreted as evidence of population-level uniformity or difference. Section 4.2 - the close correspondence of the macronutrient profile with contemporary national estimates is reported without claiming representativeness.
Funding	22	Give the source of funding and the role of the funders for the present study and, if applicable, the original study on which the present article is based.	-	Funding section - supported by USV Private Limited, Mumbai, India. The role of the sponsor, including the presence of sponsor-employed co-authors, is described in the Funding and Conflicts of Interest sections.
Ethics	-	-	nut-22.1 Describe the procedure for consent and study approval from ethics committee(s).	Section 2.2 and Institutional Review Board Statement - approved by the Institutional Ethics Committee of Shah Lifeline Hospital & Heart Institute (SLH-IEC/2025/006), approved 17 January 2025. Conducted in accordance with the Declaration of Helsinki, ICMR National Ethical Guidelines, ICH-GCP and NDCT Rules 2019. Written informed consent obtained from all participants prior to enrolment (Informed Consent Statement).

Supplementary material	-	-	nut-22.2 Provide data collection tools and data as online material, or explain how they can be accessed.	Supplementary Materials - Table S1 (this checklist); Table S2 (unadjusted and centre-adjusted differences by T2DM status); Table S3 (biochemical-data availability and characteristics according to availability); File S1 (structured dietary survey questionnaire); File S2 (data-cleaning audit trail); Figures S1 and S2. Data Availability Statement - describes conditions under which the underlying data may be accessed.

Supplementary Table S2. Unadjusted and Centre-Adjusted Differences in Primary Dietary Outcomes by T2DM Status

Dietary outcome	T2DM (n = 690), mean ± SD	Non-T2DM (n = 414), mean ± SD	Unadjusted difference (95% CI)	Model 1: age- and sex-adjusted difference (95% CI)	Model 2: fully adjusted difference (95% CI)	P value	Centre ICC
Carbohydrate (%E)	60.96 ± 5.36	64.08 ± 4.96	−3.12 (−3.75 to −2.50)	−3.23 (−3.82 to −2.63)	−2.88 (−3.41 to −2.35)	<0.001	0.133
Protein (%E)	13.13 ± 3.61	12.18 ± 3.28	0.95 (0.53 to 1.37)	0.82 (0.41 to 1.24)	0.61 (0.23 to 0.99)	<0.001	0.144
Fat (%E)	25.89 ± 5.46	23.74 ± 5.29	2.16 (1.50 to 2.81)	2.38 (1.76 to 2.99)	2.25 (1.65 to 2.85)	<0.001	0.130
Simple carbohydrate (% of total carbohydrate)	18.48 ± 5.77	22.81 ± 6.63	−4.33 (−5.10 to −3.56)	−4.15 (−4.87 to −3.43)	−4.11 (−4.83 to −3.40)	<0.001	0.143
Dietary fibre (g/day)	15.51 ± 3.17	15.15 ± 3.17	0.36 (−0.03 to 0.74)	0.28 (−0.07 to 0.64)	0.19 (−0.09 to 0.46)	0.118	0.201

Note: Values are mean ± SD. Differences are expressed as T2DM minus non-T2DM. Unadjusted differences are from Welch two-sample t-tests. Adjusted differences were estimated using linear mixed-effects models fitted by restricted maximum likelihood. Model 1 included T2DM status, age and sex as fixed effects with participating centre as a random intercept; Model 2 additionally adjusted for restricted-diet status, diet type and exercise status. P values and ICCs correspond to Model 1. Adjusted estimates describe differences between the sampled groups and do not imply an effect of T2DM on dietary intake. Abbreviations: CI, confidence interval; ICC, intraclass correlation coefficient; SD, standard deviation; T2DM, type 2 diabetes mellitus; %E, percentage of total energy intake.

Supplementary Table S3. Biochemical Data Availability and Characteristics According to Availability

Panel A. Biochemical data availability by T2DM status

Measures available	T2DM (n = 690)	Non-T2DM (n = 414)	Overall (N = 1,104)
All three measurements	562 (81.4)	0 (0.0)	562 (50.9)
Two measurements	60 (8.7)	0 (0.0)	60 (5.4)
One measurement	21 (3.0)	0 (0.0)	21 (1.9)
None	47 (6.8)	414 (100.0)	461 (41.8)
Any biochemical value	643 (93.2)	0 (0.0)	643 (58.2)

Panel B. Characteristics of participants with T2DM according to biochemical-data availability

Characteristic	Any biochemical data (n = 643)	No biochemical data (n = 47)	P value
Age (years)	51.2 ± 11.3	53.7 ± 9.3	0.084
Male sex (%)	49.5	55.3	0.532
BMI (kg/m²)	24.4 ± 3.7	23.9 ± 2.6	0.180
Restricted diet (%)	55.4	59.6	0.683
Exercise participation (%)	47.1	31.9	0.062
Vegetarian diet (%)	29.1	48.9	0.007
Energy intake (kcal/day)	1614.1 ± 276.9	1642.8 ± 326.8	0.560
Carbohydrate (%E)	60.80 ± 5.36	63.08 ± 4.90	0.003
Protein (%E)	13.14 ± 3.63	12.95 ± 3.28	0.699
Fat (%E)	26.03 ± 5.43	23.97 ± 5.48	0.016
Dietary fibre (g/day)	15.48 ± 3.17	15.97 ± 3.10	0.294
Simple carbohydrate (% of total CHO)	18.61 ± 5.81	16.68 ± 4.93	0.013

Panel C. Geographic distribution of participants with T2DM according to biochemical-data availability

Geographic zone	Any biochemical data (%)	No biochemical data (%)
North	20.8	12.8
South	34.2	6.4
East	27.8	51.1
West	17.3	29.8

Overall chi-square P < 0.001.

Note: Values are mean ± SD, n (%) or percentage, as applicable. Biochemical measures comprised fasting plasma glucose, post-prandial glucose and HbA1c obtained from available clinical records; no

study-mandated biochemical testing was performed. Panels B and C are restricted to participants with T2DM because no biochemical measurements were available for participants without T2DM. Comparisons were exploratory and intended to characterise the pattern of biochemical-data availability; no correction for multiple comparisons was applied. Continuous-variable P values in Panel B are from Welch two-sample tests and categorical-variable P values from chi-square tests. Estimated waist circumference was not included because it was not a centrally verified direct measurement. Abbreviations: BMI, body mass index; CHO, carbohydrate; HbA1c, glycated haemoglobin; SD, standard deviation; T2DM, type 2 diabetes mellitus; %E, percentage of total energy intake.

Supplementary File S1. Dietary Survey Questionnaire

Section A. General Information

1. In the last 1 year, have you followed any restricted diet?

2. Age (years)

3. Gender *

☐ Male ☐ Female ☐ Other

4. State *

Andhra Pradesh • Arunachal Pradesh • Assam • Bihar • Chhattisgarh • Delhi • Goa • Gujarat • Haryana • Himachal Pradesh • Jammu and Kashmir • Jharkhand • Karnataka • Kerala • Madhya Pradesh • Maharashtra • Manipur • Meghalaya • Mizoram • Nagaland • Odisha • Punjab • Rajasthan • Sikkim • Tamil Nadu • Telangana • Tripura • Uttar Pradesh • Uttarakhand

5. Height (cm)

6. Weight (kg)

7. Body mass index, BMI (kg/m^2)

8. Trouser size / waist circumference (inches)

9. Have you been diagnosed with Type 2 Diabetes Mellitus? *

☐ Yes ☐ No

Section B. Household Food Consumption

10. How much salt (kg) do you buy per month?

11. How many family members does this quantity serve?

12. How much oil do you buy per month (litres)?

13. How many family members does this quantity serve?

14. How much ghee do you buy per month?

15. How many family members does this quantity serve?

16. Do you use these nuts in your daily cooking? *

☐ No ☐ Peanuts ☐ Coconut ☐ Cashew nut ☐ Poppy seeds ☐ Other

17. How much quantity of the above nuts/seeds do you buy per month?

18. How many family members does this quantity serve?

Section C. Food Habits

19. What are your food habits? *

☐ Vegetarian ☐ Non-vegetarian ☐ Eggetarian ☐ Other

20. How often do you consume non-vegetarian meals (e.g., egg, chicken, fish, mutton) in a week? *

☐ Daily ☐ Once a week ☐ Thrice a week ☐ More than thrice a week ☐ Other

Section D. Dietary Recall - Day 1 (Meal Details)

21. Breakfast (Day 1). Please mention food item, quantity (1 cup = 200 mL), beverages, and sugar preference (e.g., 1) Banana = 4 2) Curd = 1 cup 3) Coffee = 1 cup [no sugar]).

22. Mid-morning snack (Day 1). Please mention food item, quantity (1 cup = 200 mL), beverages, and sugar preference.

23. Lunch (Day 1). Please mention food item, quantity (1 cup = 200 mL), beverages, and sugar preference (e.g., 1) Dal-rice = 1 cup 2) Sabji = 1/2 cup 3) Roti = 4).

24. Evening snack (Day 1). Please mention food item, quantity (1 cup = 200 mL), beverages, and sugar preference (e.g., 1) Apple = 1 2) Tea = 1 cup [no sugar]).

25. Dinner (Day 1). Please mention food item and quantity (e.g., 1) Sabji = 1 bowl 2) Roti = 4).

26. Late-night snack (Day 1). Please mention food item, quantity (1 cup = 200 mL), beverages, and sugar preference (e.g., 1) Cashew = 4 2) Milk = 1 cup [with sugar]).

27. Frequency of meals in a day *

☐ 1 ☐ 2 ☐ 3 ☐ 4 ☐ 5 ☐ 6

28. Water intake on Day 1 (litres)

Section E. Dietary Recall - Day 2 (Meal Details)

29. Breakfast (Day 2). Please mention food item, quantity (1 cup = 200 mL), beverages, and sugar preference (e.g., 1) Banana = 4 2) Curd = 1 cup 3) Coffee = 1 cup [no sugar]).

30. Mid-morning snack (Day 2). Please mention food item, quantity (1 cup = 200 mL), beverages, and sugar preference.

31. Lunch (Day 2). Please mention food item, quantity (1 cup = 200 mL), beverages, and sugar preference (e.g., 1) Dal-rice = 1 cup 2) Sabji = 1/2 cup 3) Roti = 4).

32. Evening snack (Day 2). Please mention food item, quantity (1 cup = 200 mL), beverages, and sugar preference (e.g., 1) Apple = 1 2) Tea = 1 cup [no sugar]).

33. Dinner (Day 2). Please mention food item and quantity (e.g., 1) Sabji = 1 bowl 2) Roti = 4).

34. Late-night snack (Day 2). Please mention food item, quantity (1 cup = 200 mL), beverages, and sugar preference (e.g., 1) Cashew = 4 2) Milk = 1 cup [with sugar]).

35. Frequency of meals in a day *

☐ 1 ☐ 2 ☐ 3 ☐ 4 ☐ 5 ☐ 6

36. Water intake on Day 2 (litres)

Section F. Dietary Recall - Day 3 (Meal Details)

37. Breakfast (Day 3). Please mention food item, quantity (1 cup = 200 mL), beverages, and sugar preference (e.g., 1) Banana = 4 2) Curd = 1 cup 3) Coffee = 1 cup [no sugar]).

38. Mid-morning snack (Day 3). Please mention food item, quantity (1 cup = 200 mL), beverages, and sugar preference.

39. Lunch (Day 3). Please mention food item, quantity (1 cup = 200 mL), beverages, and sugar preference (e.g., 1) Dal-rice = 1 cup 2) Sabji = 1/2 cup 3) Roti = 4).

40. Evening snack (Day 3). Please mention food item, quantity (1 cup = 200 mL), beverages, and sugar preference (e.g., 1) Apple = 1 2) Tea = 1 cup [no sugar]).

41. Dinner (Day 3). Please mention food item and quantity (e.g., 1) Sabji = 1 bowl 2) Roti = 4).

42. Late-night snack (Day 3). Please mention food item, quantity (1 cup = 200 mL), beverages, and sugar preference (e.g., 1) Cashew = 4 2) Milk = 1 cup [with sugar]).

43. Frequency of meals in a day *

☐ 1 ☐ 2 ☐ 3 ☐ 4 ☐ 5 ☐ 6

44. Water intake on Day 3 (litres)

Section G. Lifestyle and Behaviour

45. Do you exercise? *

☐ Yes ☐ No

46. If yes, type of exercise and minutes per week

47. Alcohol consumption: type of alcohol, amount (mL), and frequency in a week

48. Do you smoke or consume tobacco in any form? *

☐ Yes ☐ No

49. How many times in a week do you eat out / order food at home? *

☐ Daily ☐ Once a week ☐ Twice a week ☐ Thrice a week ☐ More than thrice a week ☐ Other

Section H. 3-Day Dietary Recall

50. Is the 3-day dietary recall completed? *

☐ Yes ☐ No

Section I. Laboratory Assessment (if available; not older than 3 months)

51. Fasting blood glucose, FBG (mg/dL)

52. Post-prandial glucose, PPG (mg/dL)

53. HbA1c (%)

54. Total cholesterol (mg/dL)

55. High-density lipoprotein cholesterol, HDL-C (mg/dL)

56. Low-density lipoprotein cholesterol, LDL-C (mg/dL)

57. Triglycerides, TG (mg/dL)

58. Additional observations / comments

** Indicates a required question.*

Supplementary File S2. Data-Cleaning Audit Trail

A source-verification audit of the analytic dataset was undertaken during preparation of the revised analysis. The rules applied are listed below and are fully reproducible from the source data file.

Rule	Scope	Change applied	Reason
R1. Energy transcription correction	3 records	Total energy corrected to source values; dependent energy fields recalculated	Factor-of-ten and digit-entry errors identified against source records
R2. Laboratory field standardisation	47 records	Unit suffixes and embedded spaces parsed to numeric values	Entries such as "280mg/dl" and "1 55" were otherwise treated as missing
R3. HbA1c unit convention	27 records (2 centres)	Values recorded as proportions rescaled to percentage units	Verified against concurrent fasting plasma glucose values
R4. Carbohydrate composition	All 1,104 records	Simple and complex carbohydrate recomputed from continuous participant-level values	Earlier calculation used pre-rounded integer fields, producing totals exceeding 100%
R5. Categorical normalisation	All records	Case normalisation of categorical variables; correction of two state-name spellings	Prevents undercounting in grouped analyses

Following correction, total energy intake ranged from 1,000 to 2,919 kcal/day and no participant fell outside the predefined plausibility range of 800–4,000 kcal/day. The analytic sample remained 1,104. Simple and complex carbohydrate proportions sum to 100% of total carbohydrate intake, and carbohydrate, protein and fat sum to 100% of total energy intake.

Supplementary Figure S1.

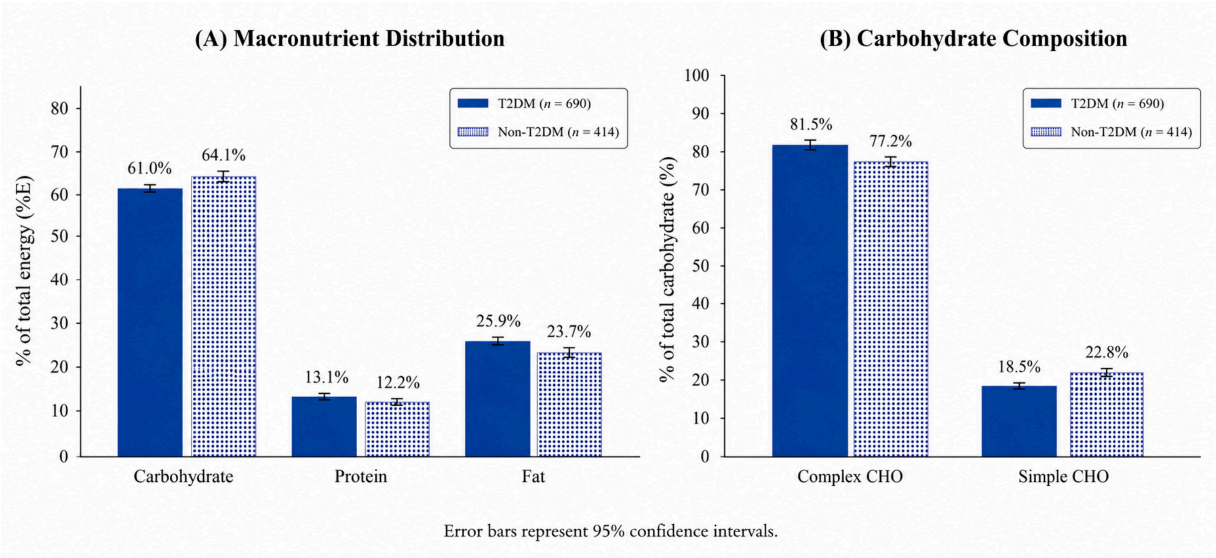

Macronutrient distribution and carbohydrate composition according to diabetes status.

(A) Mean contributions of carbohydrate, protein and fat to total energy intake among participants with and without T2DM. (B) Simple and complex carbohydrate expressed as percentages of total carbohydrate intake, by diabetes status.

Note: Values are cohort means derived from a structured three-day dietary recall. Simple carbohydrate comprises mono- and disaccharides identified at the food-code level and does not represent free-sugar intake. Abbreviations: CHO, carbohydrate; T2DM, type 2 diabetes mellitus; %E, percentage of total energy intake.

Supplementary Figure S2.

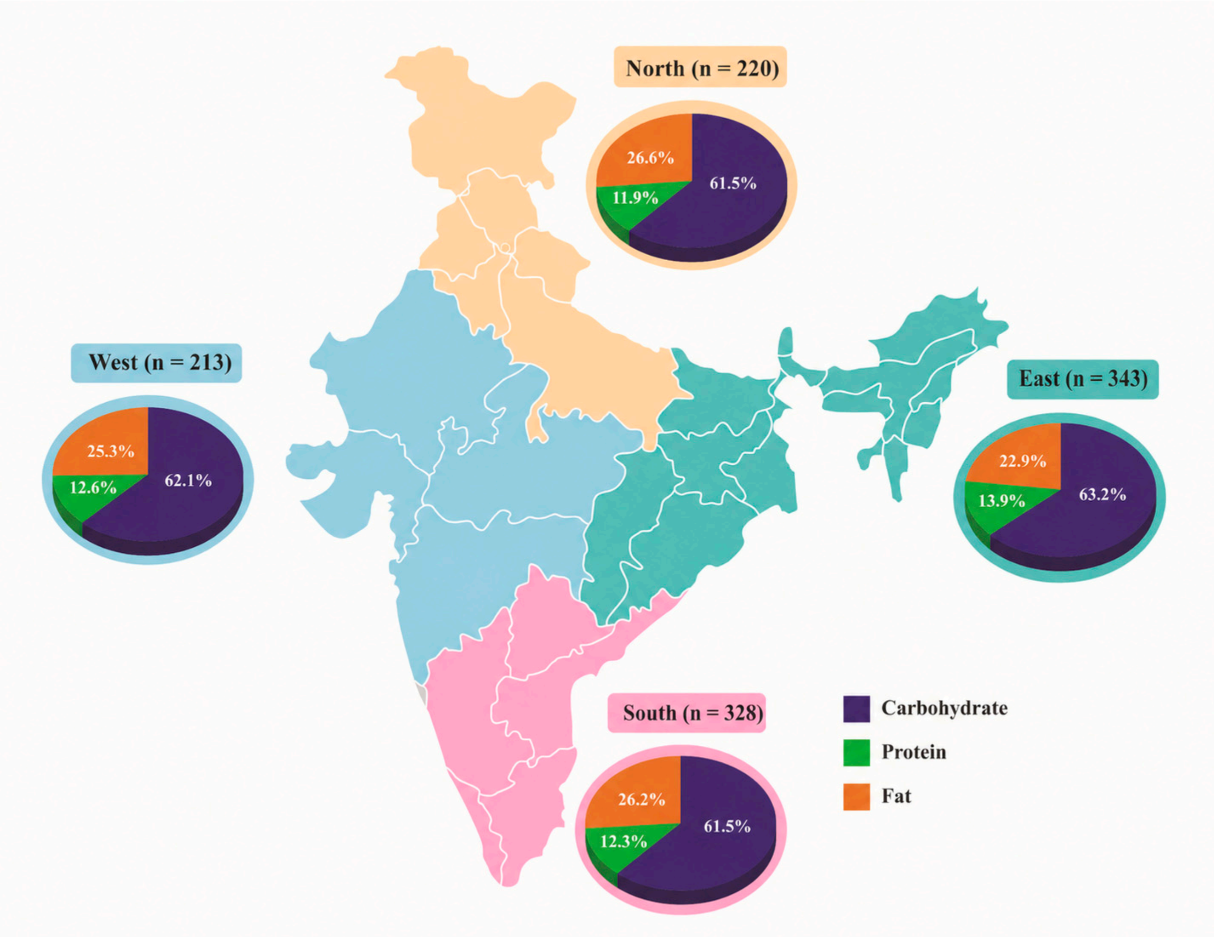

Geographic distribution of macronutrient intake across the I-STARCH-1 cohort.

Mean percentage contributions of carbohydrate, protein and fat to total energy intake across the four geographic zones (North, South, East and West).

Note: Map boundaries are illustrative and not to scale; the map is presented for descriptive purposes only. Zone comparisons were secondary and unadjusted; no correction for multiple comparisons was applied and no pairwise between-zone contrasts were tested. Abbreviations: %E, percentage of total energy intake.
